# Effect of SO_2_ on the Selective Catalytic Reduction of NO*_x_* over V_2_O_5_-CeO_2_/TiO_2_-ZrO_2_ Catalysts

**DOI:** 10.3390/ma12162534

**Published:** 2019-08-09

**Authors:** Yaping Zhang, Peng Wu, Ke Zhuang, Kai Shen, Sheng Wang, Wanqiu Guo

**Affiliations:** 1 Key Laboratory of Energy Thermal Conversion and Control of Ministry of Education, School of Energy and Environment, Southeast University, Nanjing 210096, China; 2 State Power Environmental Protection Research Institute, Nanjing 210031, China

**Keywords:** V_2_O_5_-CeO_2_/TiO_2_-ZrO_2_, selective catalyst reduction, catalyst, in situ DRIFTS, SO_2_

## Abstract

The effect of SO_2_ on the selective catalytic reduction of NO*_x_* by NH_3_ over V_2_O_5_-0.2CeO_2_/TiO_2_-ZrO_2_ catalysts was studied through catalytic activity tests and various characterization methods, like Brunner−Emmet−Teller (BET) surface measurement, X-ray diffraction (XRD), transmission electron microscopy (TEM), X-ray fluorescence (XRF), hydrogen temperature-programmed desorption (H_2_-TPR), X-ray photoelectron spectroscopy (XPS) and in situ diffused reflectance infrared Fourier transform spectroscopy (DRIFTS). The results showed that the catalyst exhibited superior SO_2_ resistance when the volume fraction of SO_2_ was below 0.02%. As the SO_2_ concentration further increased, the NO*_x_* conversion exhibited some degree of decline but could restore to the original level when stopping feeding SO_2_. The deactivation of the catalyst caused by water in the flue gas was reversible. However, when 10% H_2_O was introduced together with 0.06% SO_2_, the NO*_x_* conversion was rapidly reduced and became unrecoverable. Characterizations indicated that the specific surface area of the deactivated catalyst was significantly reduced and the redox ability was weakened, which was highly responsible for the decrease of the catalytic activity. XPS results showed that more Ce^3+^ was generated in the case of reacting with SO_2_. In situ DRIFTS results confirmed that the adsorption capacity of SO_2_ was enhanced obviously in the presence of O_2_, while the SO_2_ considerably refrained the adsorption of NH_3_. The adsorption of NO*_x_* was strengthened by SO_2_ to some extent. In addition, NH_3_ adsorption was improved after pre-adsorbed by SO_2_ + O_2_, indicating that the Ce^3+^ and more oxygen vacancy were produced.

## 1. Introduction

Selective catalytic reduction (SCR) catalysts are commonly severely deactivated by SO_2_, which is abundantly present in flue gas. There are two mechanisms that can explain the sulfur poisoning of catalysts. Firstly, SO_2_ reacts with NH_3_ and vapor in the oxygen atmosphere, producing sulfate species including ammonium sulfate and ammonium bisulfate. These sulfate substances can deposit on the catalytic surface and cause pore plugging. As a result, the specific surface area and pore volume decrease observably, ending up with the catalyst deactivation. Studies have shown that the thermal decomposition temperature range of ammonium sulfate and ammonium bisulfate are 213–308 °C and 308–419 °C [1], respectively. It is still hard for these sulfate species to decompose over a traditional V/TiO_2_ catalyst. In the second, SO_2_ can react with the active center atoms and produce metal sulfates, which will decrease catalyst activity [2,3]. The former deactivation is reversible and the catalysts can be reactivated by washing and high-temperature processing. However, the later deactivation is irreversible.

The mechanism of catalyst sulfur poisoning has been extensively studied. Wei et al. [4] claimed that SO_2_ significantly reduced the adsorption of NH_3_ on the Lewis acid sites. At the same time, SO_2_ would react with NH_4_^+^ to form NH_4_HSO_3_, thereby reducing NO*_x_* conversion. However, Jiang et al. [5] proposed that SO_2_ had little effect on the adsorption of NH_3_, and conversely promoted the formation of new Bronsted acid sites. The weakening of NO adsorption on the catalyst surface was the main cause of deactivation. With the deposition of sulfate on the catalyst surface, less NO participated in the SCR reaction, resulting in a decrease in NO*_x_* conversion. Similarly, Liu et al. [6] claimed that SO_2_ had a significant inhibitory effect on the reduction of NO*_x_* due to the deposition of sulfate substances. It hindered the adsorption of NO and the production of the intermediate ammonium nitrate, resulting in a decrease in catalyst activity. Moreover, Pan et al. [7] found that the main reason for MnO*_x_*/TiO_2_ catalysts deactivation was that the active center atom manganese was sulfated. Besides, the presence of SO_2_ caused ammonium sulfate to deposit on the catalyst, decreasing the adsorption of NO. Gu et al. [8] reported that the main reason for the deactivation of Ce/TiO_2_ catalyst was that SO_2_ could react with the catalyst to form high thermally stable Ce(SO_4_)_2_ and Ce_2_(SO_4_)_3_, and Xu et al. obtained similar results [9].

On the basis of the above literatures, it is found that there are still many controversies about the mechanism of catalyst sulfur poisoning. Moreover, there is no report found currently on the sulfur poisoning mechanism of V_2_O_5_-CeO_2_/TiO_2_-ZrO_2_ catalysts, which were reported to be an excellent SCR catalyst with a wide temperature range [10,11,12]. Our previous studies indicated that the V_2_O_5_-0.2CeO_2_/TiO_2_-ZrO_2_ catalyst exhibited superior catalytic performance as well as high tolerance of SO_2_ and H_2_O.In this paper, the catalytic activity of V_2_O_5_-0.2CeO_2_/TiO_2_-ZrO_2_ in the presence of different SO_2_ content was further studied. Various characterization methods such as the Brunner−Emmet−Teller (BET) surface measurement, X-ray diffraction (XRD), transmission electron microscopy (TEM), X-ray fluorescence (XRF), hydrogen temperature-programmed desorption (H_2_-TPR), X-ray photoelectron spectroscopy (XPS) and in situ diffused reflectance infrared Fourier transform spectroscopy (DRIFTS) were employed to study the poisoning mechanism from a microcosmic aspect. 

## 2. Materials and Methods

### 2.1. Catalyst Preparation

The Ti–Zr carrier was prepared by a coprecipitation method with the molar ratio of Ti:Zr = 1:1. An equal amount of TiCl_4_ solution and ZrOCl_2_ 8H_2_O were dissolved in deionized water and stirred constantly. With stirring, NH_3_·H_2_O was slowly added until the pH reached 10. The obtained solution was aged at room temperature for 24 h. Then, the precipitate was washed with deionized water until the supernatant contained no Cl^−^. Finally, the sample was dried at 110 °C for 12 h and then calcined at 450 °C for 4 h in a muffle furnace.

A step-by-step impregnation method was used to prepare V_2_O_5_-0.2CeO_2_/TiO_2_–ZrO_2_. A certain amount of CeNO_3_·6H_2_O and Ti–Zr powder were added to deionized water. The obtained suspension was stirred at room temperature for 2 h, followed by stirred at 85 °C for 4 h. After dried at 110 °C for 12 h and calcined in a muffle furnace at 450 °C for 4 h, the Ce/Ti–Zr sample was obtained. Thereafter, the resulting Ce/Ti–Zr was impregnated with NH_4_VO_3_ solution in the same manner. The obtained sample was denoted as V-0.2Ce/Ti–Zr, where the content of V_2_O_5_ loading was 1 wt. % and the molar ratio of Ce to Ti–Zr support = 0.2.

For short, the V-0.2Ce/Ti–Zr catalyst after reaction with 0.06% SO_2_ for 2 h was denoted as S-V-0.2Ce/Ti–Zr, while the catalyst after reaction with 10 vol% H_2_O and 0.06% SO_2_ for 2 h was denoted as HS-V-0.2Ce/Ti–Zr, respectively.

### 2.2. Activity Measurements

The SCR activity measurement of catalysts was carried out in a fixed-bed reactor with the inner diameter of 7 mm. The 0.3 g catalyst (40–60 mesh) was placed in the reactor with a gas hourly space velocity (GHSV) of 20000 h^−1^. Typically, the total gas flow was 100 mL/ min. The simulated gas was composed of 0.08% NO, 0.08% NH_3_, 5% O_2_, 5%/10% H_2_O (when used), 0.02%/0.04%/0.06% SO_2_ (when used) and N_2_ as the balanced gas. The NO, NO_2_ and NO*_x_* concentration were persistently monitored with a flue gas analyzer (Testo350-XL).

### 2.3. Catalyst Characterization

The BET was measured using a specific surface area and pore size analyzer V-Sorb 2800P (Beijing Gold APP, Beijing, China). The sample was pretreated under vacuum at 250 °C for 5 h, and the adsorbate was high purity nitrogen.

XRD patterns were carried out for phase analysis via a SmartLab™ X-ray diffractometer (Rigaku, Tokyo, Japan). Cu target acted as the X-ray source.

The morphology of the catalyst was determined by TEM (Thermo Fisher Scientific, Waltham, Massachusetts, America). The catalyst sample was dispersed in an ethanol solution after thoroughly ground, followed by shaken under ultrasonic waves for 15 min.

XRF spectrometer (Thermo Fisher Scientific, Waltham, Massachusetts, MA, America) was used to analyze the content of component in the catalyst sample. 

H_2_-TPR was carried out in a quartz U-tube reactor connected to a thermal conduction detector (TCD) using an H_2_–Ar mixture (10% H_2_ by volume) as reductant (Finetec Instruments, Hangzhou, Zhejiang, China). The temperature range during the test was for 25 °C to 800 °C with a heating rate of 10 °C /min.

XPS analysis was performed on a PHI Quantera II system (Ulvac-PHI, Chigasaki, Kanagawa Prefecture, Japan). The binding energies were referenced to the C 1 s line at 284.8 eV from adventitious carbon.

In situ DRIFTS studies were performed on a Nicolet 6700 spectrometer (Thermo Fisher Scientific, Waltham, Massachusetts, MA, USA). The scanning wave number ranged from 400 cm^−1^ to 4000 cm^−1^. Before the test, the sample was pretreated with N_2_ at 400 °C for 1 h to remove impurities. The background at a certain temperature was collected during the cooling process.

## 3. Results and Discussion

### 3.1. Effects of SO_2_ and H_2_O on Catalyst Activity

**Figure 1:**
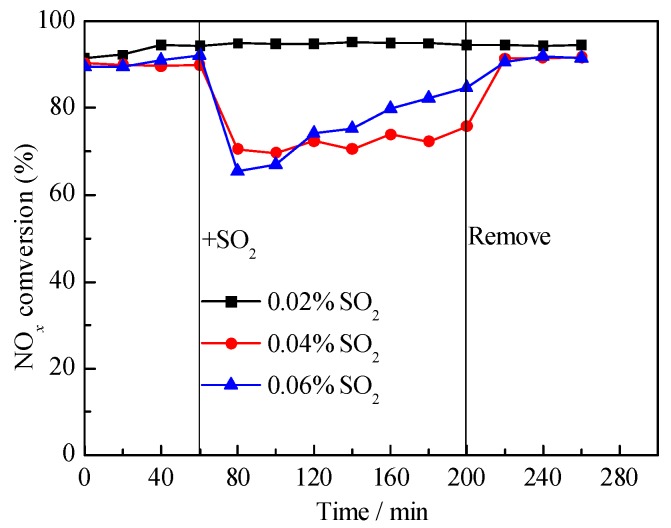
NO*_x_* conversion of V-0.2Ce/Ti–Zr in the presence of SO_2_ (250 °C). Reaction condition: NH_3_ = NO = 0.08%, O_2_ = 5%, N_2_ as balance, SO_2_ content: 0.02%, 0.04% and 0.06%, all by volume).

Figure 1 showed the catalytic activity results with different concentration SO_2_ over the V-0.2Ce/Ti–Zr catalyst at 250 °C. In the absence of SO_2_, NO*_x_* conversion of the V-0.2Ce/Ti–Zr catalyst was approximately 90%, indicating that the catalyst had high activity at the low temperature. After 0.02% SO_2_ was introduced, NO*_x_* conversion remained stable. However, NO*_x_* conversion decreased rapidly to 71% and 65% at 20 min respectively when 0.04% and 0.06% SO_2_ were added. Furthermore, it could be found that the catalytic activity could recover after stopping SO_2_. It was inferred that SO_2_ and SO_3_ reacted with NH_3_ in the early stage, leading to the decrease of NH_3_ as well as the catalytic performance. As the reaction went on, SO_2_ and SO_3_ reacted with CeO_2_ and produced Ce^3+^ in the presence of excess oxygen, which could strengthen the acid sites on the catalyst and increased NO*_x_* conversion [13].

**Figure 2:**
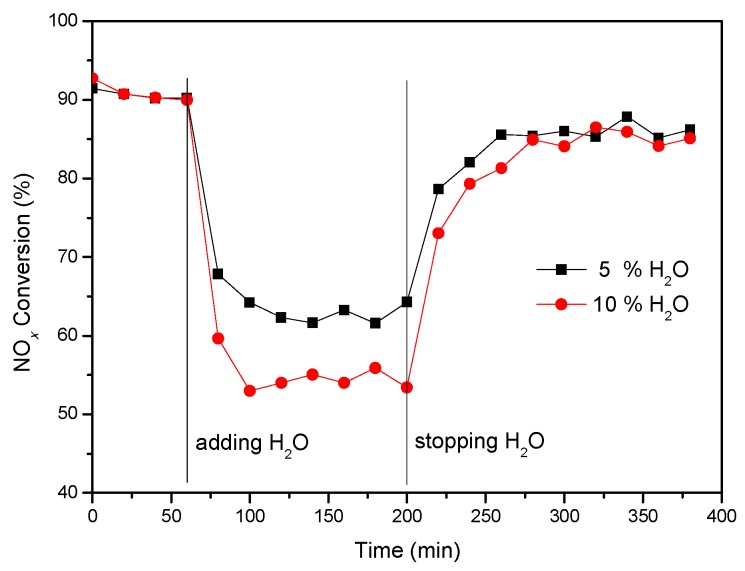
NO*_x_* conversion of V-0.2Ce/Ti–Zr in the presence of H_2_O (250 °C).

The effect of H_2_O on the catalytic activity of the catalyst was investigated and the results were shown in Figure 2. It was found that the activity of the catalyst was rapidly decreased with the introduction of H_2_O. However, after the H_2_O was stopped, the activity gradually recovered almost to the original level, thus indicating that the effect of H_2_O on the catalyst was reversible.

**Figure 3:**
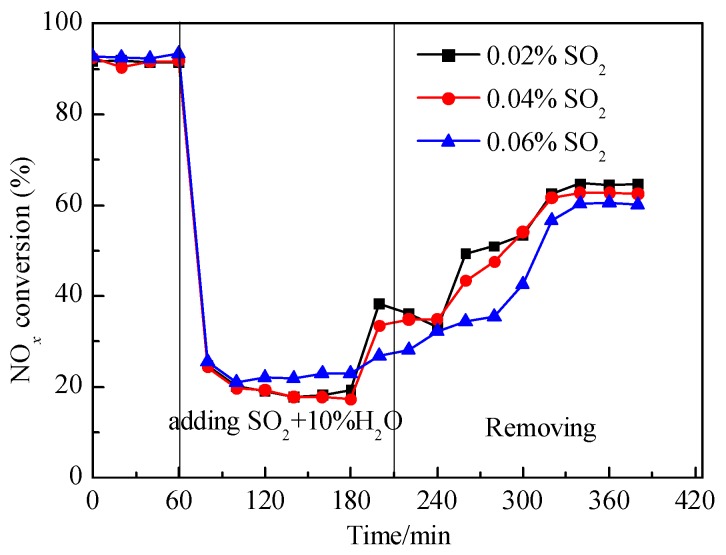
NO*_x_* conversion of V-0.2Ce/Ti–Zr in the presence of H_2_O and SO_2_ (250 °C; reaction condition: NH_3_ = NO = 0.08%, O_2_ = 5%, H_2_O = 10%, SO_2_ = 0.02%, 0.04% and 0.06%, all by volume.

The activity of the V-0.2Ce/Ti–Zr catalyst was tested at 250 °C in the presence of 10% H_2_O and different concentrations of SO_2_, and the results were shown in Figure 3. After the introduction of SO_2_ and H_2_O, NO*_x_* conversion decreased rapidly to less than 30%, which was much lower than that in the presence of SO_2_ or H_2_O alone. After SO_2_ and H_2_O were removed, NO*_x_* conversion increased to some extent but could not be restored to the initial level, indicating an irreversible deactivation occurred. Studies showed that SO_2_ would react with NH_3_ to form ammonium sulfates, and block active sites on the surface of the catalyst.

**Figure 4:**
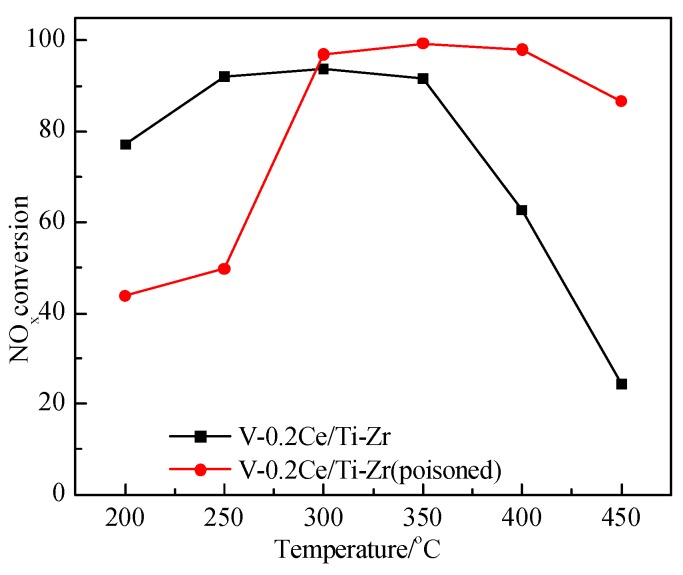
NO*_x_* conversion of the V-0.2Ce/Ti–Zr and HS-V-0.2Ce/Ti–Zr catalyst.

Furthermore, the performance of the HS-V-0.2Ce/Ti–Zr catalyst at different temperatures was also tested, and the results were shown in Figure 4. It could be found that the mid-temperature (200–300 °C) activity of the HS-V-0.2Ce/Ti–Zr catalyst was significantly decreased, while it at a higher temperature (>300 °C) was obviously enhanced. One literature indicated that the formation of Ce(SO_4_)_2_ led to a decrease in the activity of the catalyst at moderate temperatures [14]. At the same time, Xu et al. [15] pointed out that the sulphate was produced during the poisoning process, which had a certain activity at high temperatures and could improve the high temperature activity of the catalyst.

### 3.2. Physico-Chemical Characterization of Catalysts

#### 3.2.1. BET Analysis

The specific surface area of the catalysts before and after being poisoned by SO_2_ was illustrated in Table 1. Compared with the fresh sample, the BET surface area of the S-V-0.2Ce/Ti–Zr catalyst decreased from 54.45 m^2^/g to 25.07 m^2^/g, and that of the HS-V-0.2Ce/Ti–Zr catalyst further reduced to 15.27 m^2^/g. While with comparison to the fresh counterpart, there was no apparent change on the pore volume. The specific surface area of the catalyst was related to the adsorption capacity of NH_3_ during the SCR reaction, thereby further affecting the denitrification activity. 

**Figure 5:**
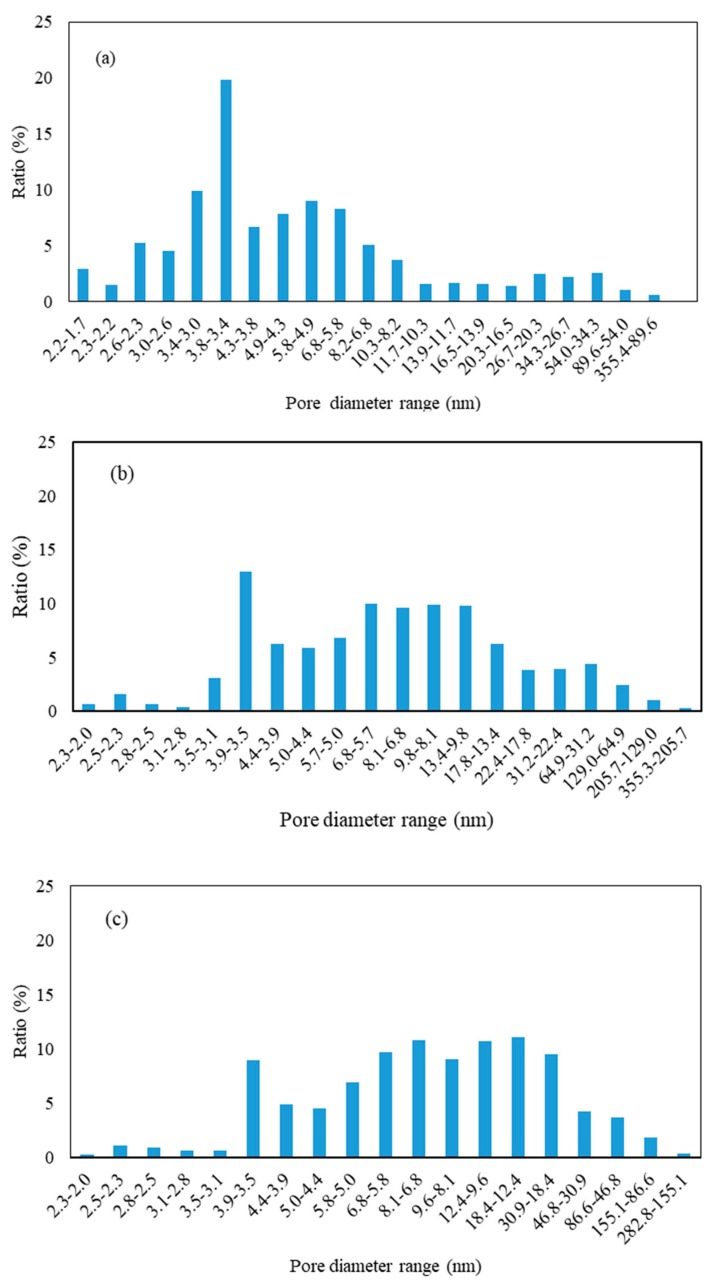
Pore size distribution of the fresh and poisoned catalysts. (**a**) V-0.2Ce/Ti–Zr; (**b**) S-V-0.2Ce/Ti–Zr and (**c**) HS-V-0.2Ce/Ti–Zr.

In order to further investigate the reason for the significant decrease in the BET specific surface area, the pore size distribution of the catalyst was mapped. As shown in Figure 5, the pore size range of the fresh catalyst was mainly concentrated at 2 nm to 10 nm, indicating that the mesopores contributed the most to the specific surface area of the catalyst. For the catalyst after reaction with O_2_, the number of macropores and mesopores (in the range of 5.3 nm to 50 nm) increased, and the increase of pore diameter in HS- V-0.2Ce/Ti–Zr catalyst was more pronounced. It was found that the mesopores (mainly 2 nm to 10 nm) contributed the most to the specific surface area of the catalyst. Therefore, it was presumed that the number of mesopores determined the NH_3_-SCR activity of the V-0.2Ce/Ti–Zr catalyst. After the reaction in presence of H_2_O and SO_2_, mesopores in the range of 5–50 nm contributed greatly to the specific surface area of the catalyst, while the mesopores and micropores with pore diameters less than 5 nm gradually decreased. Based on the above analysis, it was speculated that in the presence of SO_2_ and H_2_O, (NH_4_)_2_SO_4_ and Ce(SO_4_)_2_ formed during the reaction were adsorbed on the catalyst, and blocked 2 to 5 nm mesopores and micropores around the active component of the catalyst. These caused a decrease in specific surface area of the catalyst, further inhibiting catalytic activity.

#### 3.2.2. XRD Analysis

**Figure 6:**
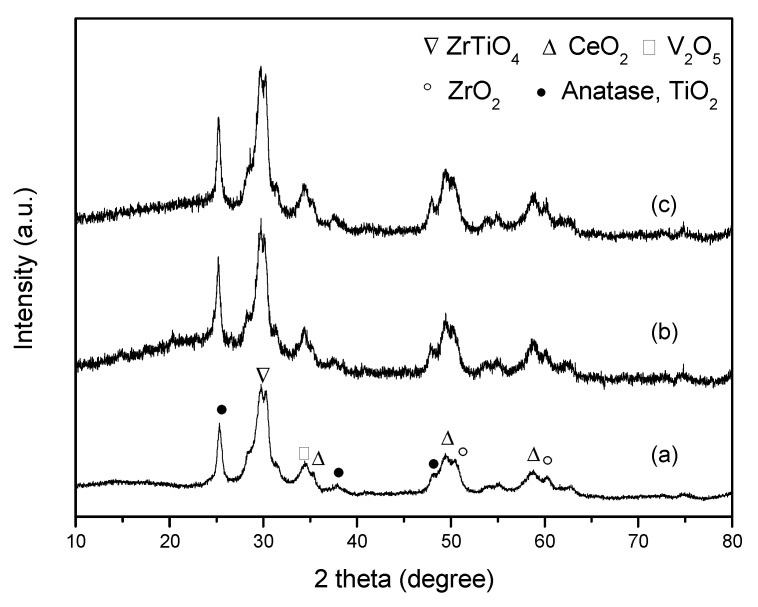
XRD patterns of the catalysts (**a**) V-0.2Ce/Ti–Zr, (**b**) S-V-0.2Ce/Ti–Zr and (**c**) HS-V-0.2Ce/Ti–Zr.

To further study the microstructure of the poisoned V-0.2Ce/Ti–Zr, an XRD analysis was performed. As shown in Figure 6, catalysts before and after being poisoned by SO_2_ showed similar spectra. According to our previous research [12], these diffraction peaks mainly corresponded to ZrTiO_2_, ZrO_2_, V_2_O_5_, CeO_2_ and TiO_2_. Diffraction peaks of substances such as (NH_4_)_2_SO_4_ or Ce(SO_4_)_2_ were not detected. The results illustrated that no sulphate with good crystal form was formed, or the amount of sulphate formed on the surface was small and highly dispersed.

#### 3.2.3. TEM and XRF Analysis

**Figure 7:**
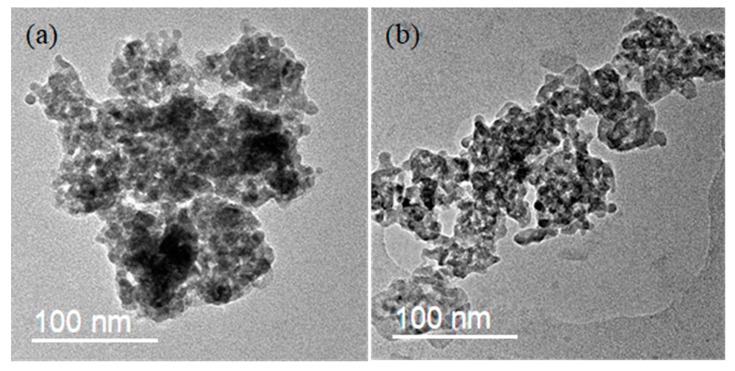
TEM pictures of the catalysts before and after poisoning by SO_2._ (**a**) V-0.2Ce/Ti–Zr and (**b**) HS-V-0.2Ce/Ti–Zr.

TEM was carried out to study the morphology changes of catalysts poisoned by H_2_O and SO_2_. As shown in Figure 7, the surface of the fresh catalyst was uniform and had a good dispersion. While for the poisoned one, it was clearly observed that the surface was covered with some substances, and the particles were agglomerate.

Element content of the catalyst was tested and the results were shown in Table 2. No S element was detected in the S-V-0.2Ce/Ti–Zr catalyst, while a trace amount of the S element was detected in the HS-V-0.2Ce/Ti–Zr catalyst. The results revealed that when SO_2_ was separately introduced, substantially no S element was present on the catalyst surface, or its content was extremely low. However, once H_2_O was introduced together, SO_2_ was more likely to participate in the reaction, and was present on the surface in the form of ammonium sulfate or Ce(SO_4_)_2_.

#### 3.2.4. H_2_-TPR Analysis

**Figure 8:**
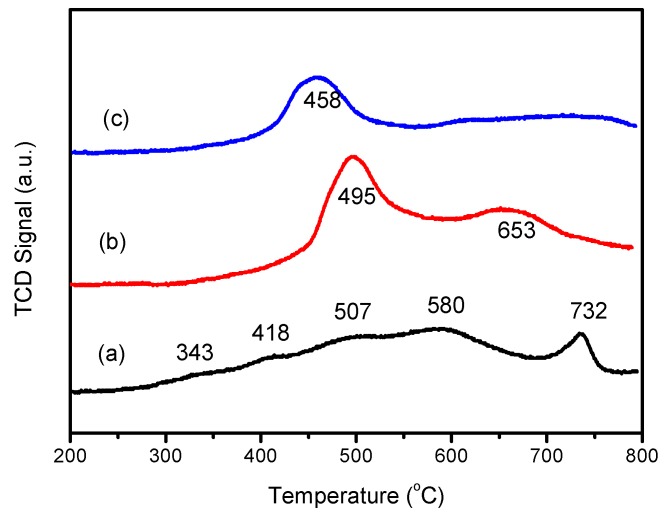
Hydrogen temperature-programmed desorption (H_2_-TPR) patterns of the catalysts before and after being poisoned by SO_2_. (**a**) V-0.2Ce/Ti–Zr, (**b**) S-V-0.2Ce/Ti–Zr and (**c**) HS-V-0.2Ce/Ti–Zr.

TPR profiles for catalysts before and after poisoned by SO_2_ were presented in Figure 8. For the fresh catalyst, five reduction peaks were observed at 343 °C, 418 °C, 507 °C, 580 °C and 732 °C, respectively. Studies have shown that the low temperature reduction of V/TiO_2_ catalyst is mainly related to the monomer vanadium or highly dispersed vanadium species [16]. Held et al. proposed that the reduction peak at about 730 °C was mainly related to V_2_O_4_ [17]. Based upon our previous work, peaks centered at 343 °C and 580 °C were due to the reduction of vanadium from V^5+^ to V^4+^ and V^4+^ to V^3+^, respectively. The reduction peak centered at 418 °C was attributed to the surface (α) reduction processed of CeO_2_, and the subsurface layers and deeper regions of the catalyst nanoparticles were reduced at 507 °C.

It could be found that the redox ability of the S-V-0.2Ce/Ti–Zr catalyst was significantly weakened, according to the fact that several peaks disappeared (343 °C, 418 °C and 580 °C). After reaction with SO_2_ and H_2_O, the redox ability of the HS-V-0.2Ce/Ti–Zr catalyst was further attenuated, and only one reduction peak appeared at 458 °C was detected. Peaks centered at 495 °C and 458 °C were due to the reduction of Ce^4+^, while the peak at 653 °C was attributed to the reduction of V^5+^ to V^3+^ and the reduction of bulk phase Ce^4+^. It could be found that the disappearance of reduction peaks related with vanadium was an important cause of catalytic deactivation. For S-V-0.2Ce/Ti–Zr sample, the α-reduction peak of Ce^4+^ disappeared, which should be related to the reduction of Ce^4+^ to Ce^3+^. It was reported that the concentration of Ce^3+^ was used to assess the amount of oxygen formed. The oxygen vacancies were driven by the transition from Ce^4+^ to Ce^3+^, accelerating the transport of active oxygen species and facilitating the reaction vacancies, which could explain the phenomenon that the activity of the catalyst decreased first and then gradually increased. After being poisoned by SO_2_ and H_2_O, the intensity of the β reduction peak (458 °C) was significantly weakened, and the reduction peak of the vanadium oxide completely disappeared. It was speculated that the formed ammonium hydrogen sulfate was deposited on the surface of the catalyst or more stable Ce_2_(SO_4_)_3_ was formed, which hindered the conversion of vanadium active species and led to a reduction of the redox ability. 

#### 3.2.5. XPS Analysis

**Figure 9:**
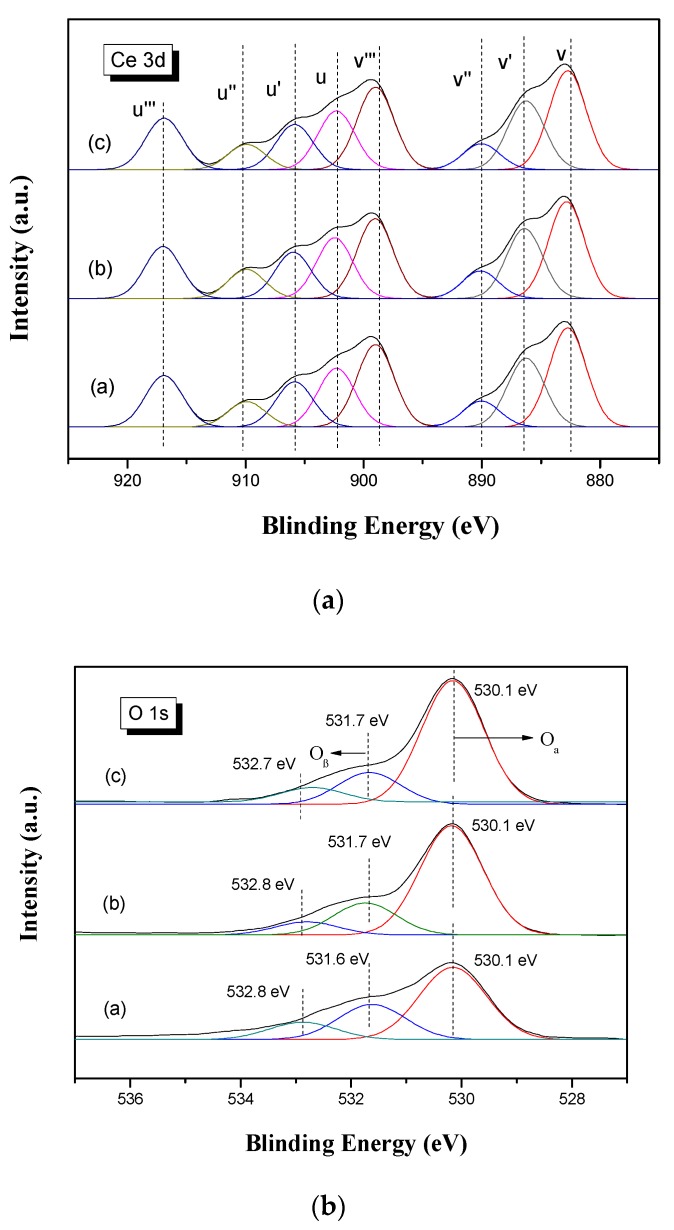
XPS spectra of Ce 3d and O 1s on catalysts before and after being poisoned by SO_2_. (**a**) V-0.2Ce/Ti–Zr; (**b**) S-V-0.2Ce/Ti–Zr and (**c**) HS-V-0.2Ce/Ti–Zr.

Figure 9a showed the XPS results of Ce 3d. Peaks u′′′, u′′, u and v′′′, v′′, v can be corresponded to Ce^4+^, while peaks u′ and v′ were assigned to Ce^3+^, respectively. It can be seen from Figure 9a that Ce in the catalyst mainly existed in the Ce^4+^ valence state. As shown in Table 3, after reacting with 0.06% SO_2_ for 2 h, the ratio of Ce^3+^/ (Ce^4+^+Ce^3+^) in S-V-0.2Ce/Ti–Zr catalysts was increased to 25.15% compared with that in V-0.2Ce/Ti–Zr (24.94%), indicating more Ce^3+^ was generated. However, in the presence of SO_2_ and H_2_O, the ratio of Ce^3+^/(Ce^4+^ + Ce^3+^) was reduced again to 24.93%. Hence, it was deduced that the introduction of SO_2_ promoted the conversion of Ce^4+^ and Ce^3+^, while the formed ammonium hydrogen sulfate on the surface of the catalyst could block the path of mutual conversion between Ce^4+^ and Ce^3+^.

As shown in Figure 9b, the three peaks in O 1 s can be assigned to oxygen vacancy, adsorbed oxygen and oxygen lattice from higher binding energy to lower binding energy. The adsorbed oxygen and oxygen lattice were marked as Oβ and Oα, respectively [18,19]. The oxygen vacancy was formed due to the evolution of the oxygen lattice [20].

As can be seen in Table 3, the ratio of Oβ/(Oβ + Oα) was reduced significantly in the S-V-0.2Ce/Ti–Zr catalysts and even worse in the HS-V-0.2Ce/Ti–Zr catalysts. The existence of Oβ could promote the oxidation of NO to NO_2_, which can explain the decrease in activity of the catalysts poisoned by SO_2_.

### 3.3. In Situ DRIFTS Study

#### 3.3.1. Sulfur Dioxide Adsorption.

**Figure 10:**
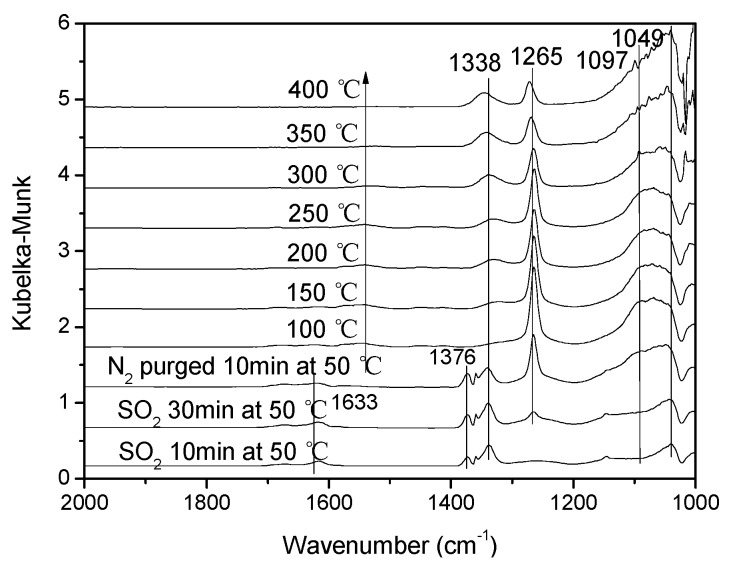
In situ DRIFTS spectra of V-0.2Ce/Ti–Zr treated in flowing 0.06% SO_2_ at 50 °C and then purged by N_2_ at a different temperature.

Figure 10 showed the DRIFTS spectra of the V-0.2Ce/Ti–Zr catalyst in the flow of 0.06% SO_2_ at 50 °C and then purged by N_2_ with increasing temperatures from 50 °C to 400 °C. The peak at 1633 cm^−1^ was linked to H_2_O vibration produced by the reaction of SO_2_ and hydroxyl on the catalytic surface [8]. With the addition of SO_2_ at 50 °C, peaks at 1376 cm^−1^, 1338 cm^−1^, 1265 cm^−1^, 1097 cm^−1^ and 1049 cm^−1^ were detected. Based on the research of Peak et al. [21], the triply degenerate asymmetric stretching ν3 band were accessible to FTIR investigation, and would split into three bands when the bidentate sulfate complex was formed. Therefore, it was deduced that the bands at 1265 cm^−1^, 1097 cm^−1^ and 1049 cm^−1^ were attributed to bidentate sulfate on V-0.2Ce/Ti–Zr. The band at 1338 cm^–1^ was assigned to the adsorbed SO_2_, which mainly exists in the form of SO_3_^2−^. The band at 1376 cm^−1^ might be due to the asymmetric vibration of O = S = O covalent groups (SO_4_^2–^). When the temperature reached 100 °C, the adsorption peak at 1376 cm^−1^ disappeared [22,23]. 

**Figure 11:**
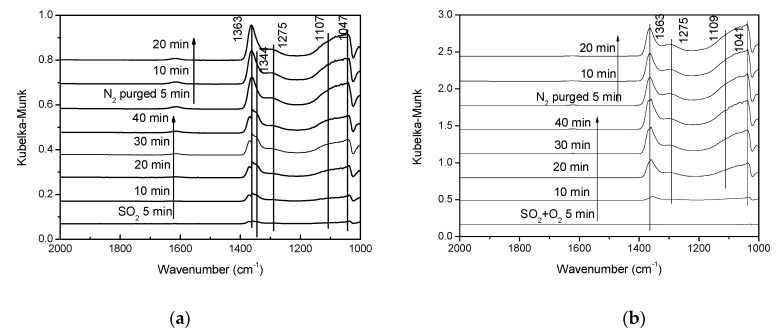
In situ DRIFTS spectra recorded at 250 °C in a flow of 0.06% SO_2_ and 0.06% SO_2_ + 5% O_2_ over V-0.2Ce/Ti–Zr. (**a**) SO_2_ adsorption and (**b**) SO_2_ + O_2_ co-adsorption.

Figure 11a shows the in situ DRIFTS spectra of the V-0.2Ce/Ti–Zr catalyst in the flow of 0.06% SO_2_ at 250 °C and then purged with N_2_. After adding SO_2_ for 5 min, bands at 1363 cm^−1^, 1344 cm^−1^, 1295 cm^−1^, 1083 cm^−1^ and 1047 cm^−1^ were detected with increasing intensity. The bands at 1295 cm^−1^, 1107 cm^−1^ and 1050 cm^−1^ were contributed to the bidentate sulfate on V-0.2Ce/Ti–Zr while the band at 1344 cm^–1^ was assigned to the adsorbed SO_2_ (SO_3_^2–^). The band at 1363 cm^−1^ could be attributed to the asymmetric vibration of O = S = O covalent groups (SO_4_^2–^). It had been found that the O = S = O asymmetric covalent groups arose from the VOSO_4_ adsorption peak that appeared at 1383 cm^−1^ [24,25]. Hence, we deduced the reaction between the adsorbed SO_2_ with V_2_O_5_ in the catalyst to form the VOSO_4_ intermediate.

Figure 11b shows the DRIFTS spectra of the V-0.2Ce/Ti–Zr catalyst in the flow of O_2_ + SO_2_ at 250 °C and then purged with N_2_. In general, catalysts exhibited similar peaks, while the intensity of SO_2_ adsorption was enhanced significantly in the presence of O_2_.

#### 3.3.2. Effect of SO_2_ on NH_3_ Adsorption

**Figure 12:**
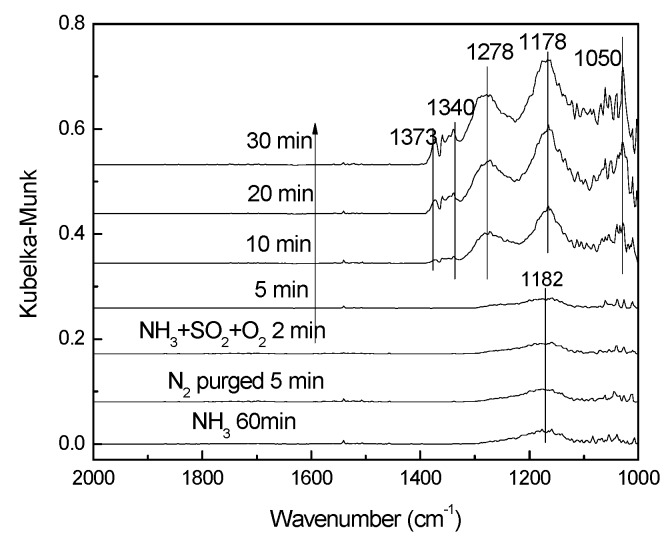
In situ DRIFTS spectra of V-0.2Ce/Ti–Zr pretreated by NH_3_ exposed to NH_3_ + SO_2_ + O_2_ for various times.

Figure 12 showed the NH_3_ adsorption results on V-0.2Ce/Ti–Zr in the presence of SO_2_. It could be found that original adsorption capacity of NH_3_ was very weak, and only the band at 1182 cm^−1^ was observed [26]. Then NH_3_ was switched off and SO_2_ + O_2_ was introduced. The NH_3_ adsorption peak was replaced by SO_4_^2−^ adsorption peak quickly. Bands at 1278 cm^−1^, 1178 cm^−1^ and 1050 cm^−1^ were assigned to SO_4_^2−^ three-fold degeneracy asymmetric stretching vibration v3 and the band at 1340 cm^−1^ was assigned to the adsorbed SO_2_ while the band at 1373 cm^−1^ could be attributed to the asymmetric vibration of O = S = O covalent groups (SO_4_^2–^).

**Figure 13:**
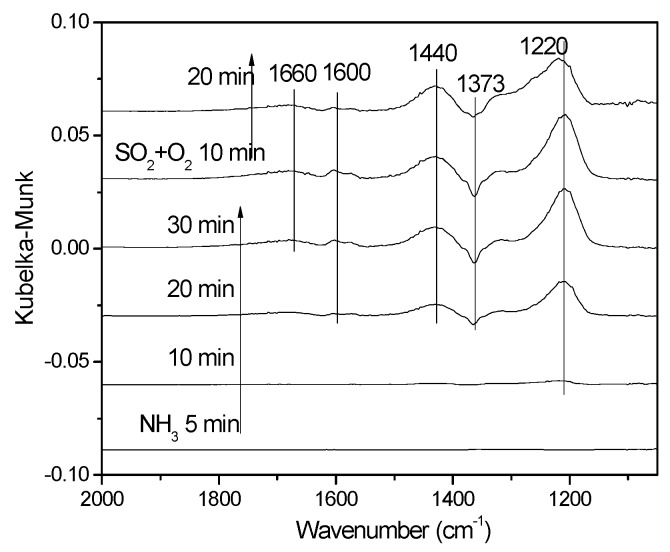
In situ DRIFTs spectra of V-0.2Ce/Ti–Zr pretreated by SO_2_ + O_2_ exposed to NH_3_ for various times and then treated by SO_2_ + O_2_ again.

In Figure 13, the catalysts were pretreated by 0.06% SO_2_ + O_2_ for 30 min, and then exposed to 800 ppm NH_3_, followed by treating with SO_2_ and O_2_ again. After adding NH_3_, several bands at 1660 cm^−1^, 1600 cm^−1^, 1440 cm^−1^ and 1220 cm^−1^ were detected. The band at 1600 cm^−1^ and 1220 cm^−1^ was associated with the asymmetric and symmetric deformation vibration of NH_3_ adsorbed on Lewis acid sites, while the band at 1660 cm^−1^ and 1440 cm^−1^ were due to the asymmetric and symmetric deformation of NH_4_^+^ bound to Brönsted acid sites [26,27]. Compared with the results of Figure 9, it could be found that the intensity of bands due to adsorbed NH_3_ was significantly increased after being pretreated by SO_2_ and O_2_. It was deduced that the presence of SO_2_ and O_2_ could promote the transformation from Ce^4+^ to Ce^3+^ (the formation of Ce_2_(SO_4_)_3_), resulting in the enhancement of the NH_3_ adsorption ability. Furthermore, it was observed that the intensity of the NH_3_ adsorption peaks did not change significantly after the introduction of SO_2_ and O_2_ again.

#### 3.3.3. Effect of SO_2_ on NO Adsorption

**Figure 14:**
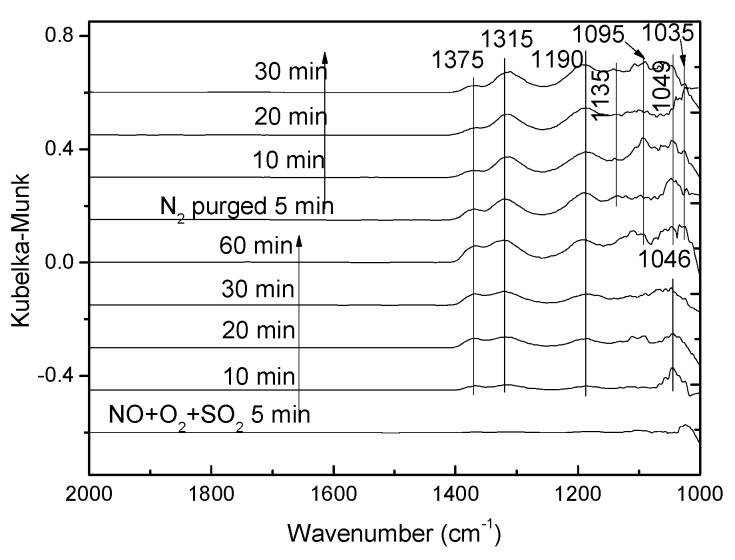
In situ DRIFTS spectra taken at 250 °C in a flow of 0.08% NO + 0.06% SO_2_ + 5% O_2_ on V-0.2Ce/Ti–Zr.

**Figure 15:**
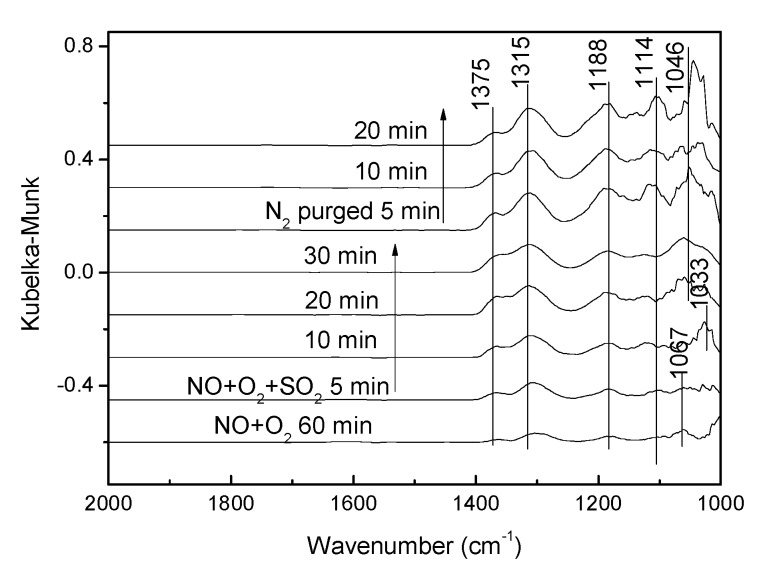
In situ DRIFTS spectra taken at 250 °C in a flow of 0.08% NO + 0.06% SO_2_ + 5% O_2_ over the NO + O_2_ presorbed on V-0.2Ce/Ti–Zr.

The competitive adsorption behavior of SO_2_ with NO_2_ in the presence of O_2_ was investigated, and in situ DRIFTS results were shown in Figure 14. As shown in Figure 15, bands appeared at 1375 cm^−1^ and 1315 cm^−1^ were due to cis-N_2_O_2_^2−^, and it could be found that the intensity of the bands increased gradually and the purge of N_2_ had little influence on the adsorption bands. Band at 1190 cm^−1^ were attributed to the bridging nitrates [28]. After 10 min, SO_4_^2−^ adsorption peak appeared at 1046 cm^−1^. As the reaction went on, it divided into two adsorption peaks (1049 cm^−1^ and 1035 cm^−1^). Along with the purge of N_2_, band at 1095 cm^−1^ assigned to bidentate sulfate was observed. Furthermore, weak SO_4_^2−^ adsorption band was detected at 1135 cm^−1^. 

In Figure 15, the catalyst was first treated by NO + O_2_ for 60 min until the adsorption was saturated and 0.06% SO_2_ was added into the cell, and then SO_2_ were introduced into the in-situ cell for various times, followed by N_2_ purging. As shown in Figure 15, bands due to the cis-N_2_O_2_^2−^ were also detected at 1375 cm^−1^ and 1315 cm^−1^. Band at 1188 cm^−1^ was assigned to the adsorbed NO*_x_* species. Nitrate species adsorption peak appeared at 1144 cm^−1^ and 1067 cm^−1^ after NO + O_2_ pre-adsorption. Once feeding SO_2_, the intensity of the bands at 1375 cm^−1^, 1315 cm^−1^, 1188 cm^−1^ and 1144 cm^−1^ were strengthened and the purge of N_2_ had little influence on the adsorption bands, while the band at 1067 cm^−1^ gradually disappeared. The SO_4_^2−^ adsorption peak appeared at 1046 cm^−1^.

Based on the analysis above, it could be concluded that the presence of SO_2_ could promote NO adsorption on the catalyst surface. In addition, our previous research has a certified catalyst that mainly follows the Eley–Rideal (E-R) mechanism, and NO adsorption on the catalyst surface restrains the SCR reaction [12]. Thus, it can explain the decrease of catalyst activity when SO_2_ was injected.

#### 3.3.4. Effect of SO_2_ on NH_3_ + NO + O_2_ Co-Adsorption 

**Figure 16:**
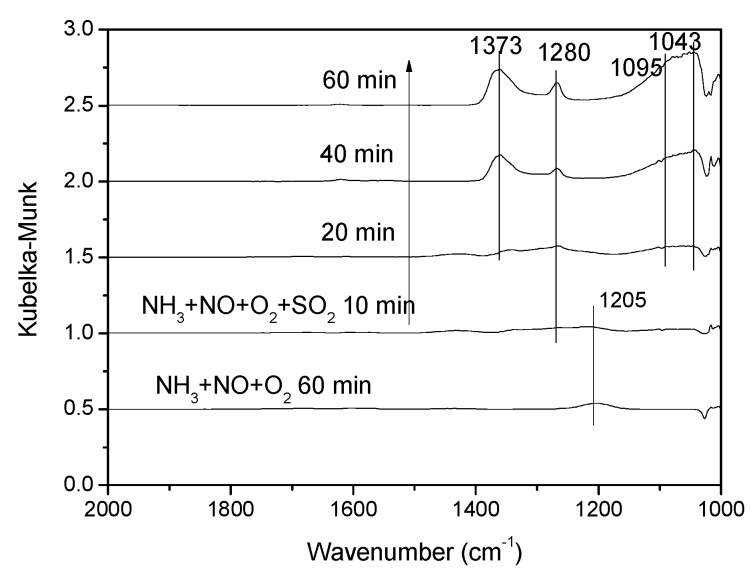
In situ DRIFTS spectra of the NH_3_ + NO + O_2_ reaction on V-0.2Ce/Ti–Zr (250 °C) with 0.06% SO_2._

As shown in Figure 16, in the presence of NH_3_ + NO + O_2_, only the band at 1205 cm^−1^ due to adsorbed NH_3_ was observed. After 0.06% SO_2_ was injected, adsorption intensity of bands due to adsorbed NH_3_ decreased rapidly. These results indicated that the presence of SO_2_ significantly weakened the NH_3_ adsorption, which was consistent with the result of Figure 12. After the introduction of SO_2_ for 20 min, bands at 1373 cm^−1^, 1280 cm^−1^, 1095 cm^−1^ and 1043 cm^−1^ were detected. The band at 1373 cm^−1^ could be attributed to the asymmetric vibration of O = S = O covalent groups and bands at 1280 cm^−1^, 1095 cm^−1^ and 1043 cm^−1^ were assigned to SO_4_^2−^ three-fold degeneracy asymmetric stretching vibration v3. In our previous study [12], the SCR reaction over V-0.2Ce/Ti–Zr obeyed the E-R mechanism. Hence, as an important reaction substance, the weakening of NH_3_ adsorption would greatly inhibit the SCR reaction.

### 3.4. Possible SO_2_ Reaction Mechanism Over The Catalyst

The adsorption ability of SO_2_ had something with its concentration. There was no adsorption when SO_2_ concentration was low, which explained the stable activity of catalysts at low SO_2_ concentration. With the increase of the SO_2_ concentration, SO_2_ and SO_4_^2−^ adsorption appeared. SO_2_ was adsorbed on the surface in the form of SO_3_^2^^−^. The consumption of surface OH species meant that SO_2_ was able to react with surface hydroxyl groups to form adsorbed H_2_O. It was speculated that in the presence of O_2_, SO_2_ would react with NH_3_ to form (NH_4_)_2_SO_4_, which could deposit on the surface of the catalyst and reduce the catalyst activity. The sulfate products could combine with Ce to form more stable Ce(SO_4_)_2_. The reaction might be proposed as follows:

SO_2_(g) + O^2−^(a) →SO_3_^2−^(a), (1)

SO_2_(g) + 2OH^−^(a) →SO_3_^2−^ (a) + H_2_O, (2)

O_2_(g) →2[O](a), (3)

SO_3_^2−^ (a) + [O](a) →SO_4_^2−^ (a) (4)

SO_4_^2−^ (a) + 2NH_4_^+^(a)→(NH_4_)_2_SO_4_ (5)

Ce^4+^ + SO_4_^2−^ →Ce(SO_4_)_2_ (6)

According to the in-situ DRIFTS results and other study [19], SO_2_(SO_3_^2–^) could react with V^5+^–OH to form VOSO_4_ intermediate.

SO_3_^2−^ (a) + V^5+^-OH(a) →VOSO_4_ (7)

With the time passing, absorbed SO_2_ (SO_3_^2−^) contacted with the active component Ce and caused the transformation from Ce^4+^ to Ce^3+^, increasing the oxygen vacancy and enhancing the NH_3_ adsorption ability, which can explain the recovery of activity. The presence of O_2_ promoted the redox of SO_2_ and formation of sulfate Ce_2_(SO_4_)_3_.

3SO_2_ + 2CeO_2_ + O_2_ →Ce_2_(SO_4_)_3_ (8)

## 4. Conclusions

Catalyst activity test results showed that low concentration SO_2_ (<0.02%) had little influence on catalyst activity. With increasing SO_2_ concentration, NO*_x_* conversion of the catalyst gradually decreased but could restore to the original level when stopping feeding SO_2_. A reversible deactivation would occur in the presence of H_2_O. However, in the presence of SO_2_ and H_2_O, the catalyst was irreversibly deactivated, which was worse than that of adding SO_2_ or H_2_O alone.

The characterization results showed that the BET specific surface area of the catalysts poisoned by SO_2_ were reduced, and the redox capacity were significantly weakened. The sulfuration of the active component Ce and the deposition of ammonium sulfate might be the causes of the deactivation. XPS analysis showed that the presence of SO_2_ promoted the generation of Ce^3+^, which probably promoted the SCR reaction.

In situ DRIFTS analysis indicated that the adsorption capacity of SO_2_ was enhanced in the presence of O_2_. The presence of SO_2_ would inhibit the adsorption of NH_3_. At the same time, the NO adsorption ability of the catalyst was enhanced to some extent by SO_2_. Moreover, the NH_3_ adsorption ability of the catalyst with pre-adsorption of SO_2_ + O_2_ was markedly enhanced, indicating that when SO_2_ + O_2_ existed, more Ce^3+^ and oxygen vacancy were produced. These conclusions might contribute to a better understanding of the SO_2_ poisoning mechanism over the V_2_O_5_–CeO_2_/TiO_2_–ZrO_2_ catalyst.

## Figures and Tables

**Table 1:** BET surface area and pore volume of the fresh and poisoned catalyst.

Sample	BET Surface Area (m^2^/g)	Pore Volume (mL/g)
V-0.2Ce/Ti–Zr	54.45	0.14
S-V-0.2Ce/Ti–Zr	25.07	0.16
HS-V-0.2Ce/Ti–Zr	15.27	0.11

**Table 2:** XRF results of the catalysts before and after being poisoned by SO_2_.

Sample	V	Ce	Ti	Zr	S
V-0.2Ce/Ti–Zr	0.723	12.512	1.2	34.66	0
S-V-0.2Ce/Ti–Zr	0.63	12.365	19.75	26.66	0
HS-V-0.2Ce/Ti–Zr	0.558	12.932	20.46	26.4	1.13

**Table 3:** Peak area ratio of XPS.

	Area Ratio	
Sample	$\frac{Ce^{3+}}{Ce^{3+}+Ce^{4+}}$	$\frac{O_{\beta }}{O_{\beta }+O_{\alpha }}$
V-0.2Ce/Ti–Zr	24.94	28.14
S-V-0.2Ce/Ti–Zr	25.15	20.71
HS-V-0.2Ce/Ti–Zr	24.93	18.67
